# Evaluation of Muscle Mass and Malnutrition in Patients with Colorectal Cancer Using the Global Leadership Initiative on Malnutrition Criteria and Comparing Bioelectrical Impedance Analysis and Computed Tomography Measurements

**DOI:** 10.3390/nu16173035

**Published:** 2024-09-08

**Authors:** Daniel de Luis Roman, Juan José López Gómez, Marife Muñoz, David Primo, Olatz Izaola, Israel Sánchez

**Affiliations:** 1 Centro de Investigación of Endocrinología and Nutrición, Facultad de Medicina, Universidad de Valladolid, 47003 Valladolid, Spain; 2 Departamento de Endocrinología y Nutricion, Hospital Clinico Universitario de Valladolid, 47003 Valladolid, Spain; 3 Unidad de Apoyo a la Investigación, Hospital Clínico Universitario de Valladolid, 47003 Valladolid, Spain; 4 Svo. Radiología, Hospital Clínico Universitario de Valladolid, 47003 Valladolid, Spain

**Keywords:** cancer colorectal, low muscle mass, malnutrition, SMI-BIA, SMI-CT

## Abstract

Objectives: The aim of this investigation was to evaluate the discrepancies between bioelectrical impedance analysis (BIA) and computed tomography (CT) in assessing skeletal muscle mass and identifying low muscle mass in patients with colorectal cancer. Methods: This study recruited 137 patients with colorectal cancer from February 2028 to December 2023. CT scans were analyzed at the Lumbar 3 vertebral level to determine the area of skeletal muscle, which was then utilized to estimate whole-body skeletal muscle mass. [BIA] was also employed to measure skeletal muscle. Both skeletal muscle mass values [kg] were divided by height^2^ [m^2^] to calculate the skeletal muscle index [SMI, kg/m^2^], denoted as SMI-CT and SMI-BIA, respectively. Results: The median age was 69.8 + 9.5 years, with the sex ratio being 88/49 [male/female]. Whereas more than one-third of the patients were classified as malnourished based on the Global Leadership Initiative on Malnutrition GLIM-CT criteria using L3-SMI [*n* = 36.5%], fewer patients were classified as malnourished based on GLIM-BIA using SMI-BIA [*n* = 19.0%]. According to the CT analysis [low SMI-L3], 52 [38.0%] patients were diagnosed as having poor muscle mass, whereas only 18 [13.1%] patients were identified as having low muscle mass using BIA [low SMIBIA]. The measured SMI showed a positive association with SMI-CT in all patients [r = 0.63, *p* < 0.001]. Using Bland–Altman evaluation, a significant mean bias of 0.45 + 1.41 kg/m^2^ [95% CI 0.21–0.70; *p* < 0.001] between SMI-BIA and SMI-CT was reported. Receiver operating characteristic (ROC) curves were generated to detect poor muscle mass using SMI-BIA with CT as the gold standard. The area under the curve (AUC) for SMI-BIA in identifying poor muscle mass was 0.714 (95% CI: 0.624–0.824), with a good cut-off value of 8.1 kg/m^2^, yielding a sensitivity of 68.3% and a specificity of 66.9%. Conclusions: BIA generally overestimates skeletal muscle mass in colorectal cancer patients when contrasted to CT. As a result, BIA may underestimate the prevalence of poor muscle mass and malnutrition according to the GLIM criteria in this patient population.

## 1. Introduction

Poor muscle mass has been identified in approximately 30% of patients at the time of cancer diagnosis and is linked to negative clinical outcomes, such as significantly longer post-operative hospital stays, a heightened risk of postoperative complications, along with reduced overall and disease-free survival rates [1]. In accordance with the Global Leadership Initiative on Malnutrition [GLIM] criteria, poor muscle mass is recognized as a primary phenotypic criterion for diagnosing malnutrition [2]. The GLIM Body Composition Working Group has underscored the essential importance of muscle mass evaluation in clinical settings to ensure more accurate diagnosis of malnutrition and to enhance overall clinical outcomes [3].

Currently, both magnetic resonance imaging (MRI) and computed tomography (CT) are considered the reference standard evaluations for non-invasive muscle mass evaluation [4,5]. In particular, measuring the cross-sectional area at the level of the Lumbar 3 vertebra (L3) using CT is recognized as a representative marker for skeletal muscle in real-world evaluations [4,5]. Despite the high specificity and accuracy of CT scan analysis, this technique presents certain limitations, including the lack of portability, high cost, exposure to ionizing radiation, and technical complexity [6]. Bioelectrical impedance analysis [BIA], on the other hand, is a straightforward, non-invasive, and low-cost technique for assessing body composition, which is suitable for use with patients in a wide range of settings [7]. BIA indirectly estimates body composition partitions, such as fat-free mass, fat mass, and skeletal muscle mass, using formulas based on electrical parameters. However, these formulas have primarily been validated in healthy populations, which may limit their accuracy in assessing body composition in cancer patients—a group with a high prevalence of disease-related malnutrition [DRM] [8]. Furthermore, cancer patients are more prone to DRM and alterations in hydration status, which can impact the reliability of BIA in assessing body composition. As a result, the use of BIA for determining skeletal muscle mass in cancer patients within clinical settings remains uncertain.

Several studies have shown a strong correlation between skeletal muscle mass measurements obtained by CT and BIA in oncological patients, including those with cancer and disease-related malnutrition [DRM] [9]. However, despite this high correlation, BIA often overestimates skeletal muscle mass in certain patients in contrast to CT [10], which could lead to an underestimation of DRM diagnosis, given that muscle mass is one phenotypic criterion for this condition [2]. Nevertheless, there are limited data comparing CT and BIA methods in assessing skeletal muscle mass and diagnosing malnutrition in accordance with the GLIM criteria in patients with colorectal cancer.

The aim of this study was to evaluate the discrepancies between CT and BIA in assessing skeletal muscle mass and identifying poor muscle mass in patients with colorectal cancer, and their usefulness in making a correct DRM diagnosis based on the GLIM criteria.

## 2. Materials and Methods

### 2.1. Patients

Our study involved 137 patients with colorectal cancer without metastasis, including an Eastern Cooperative Oncology Group [ECOG] performance status of 0 or 1, who were sent to the Department of Nutrition at the Hospital Clinic Universitario de Valladolid for nutritional evaluation. The exclusion criteria included the following: (1) ineligibility for bioelectrical impedance analysis (BIA) due to conditions like metallic implants, pacemakers, or limb amputations; (2) lack of abdominal CT scans performed at our institution within the past month; (3) the presence of other concurrent malignant tumors, heart attack, renal chronic disease, or liver chronic disease.; or (4) prior neo-adjuvant chemotherapy. The protocol [code PIP24-264] was authorized by the Ethics Committee for Research of the Health Council of HCUVA. All patients participating in this study gave informed consent and STROBE guides were followed (Supplementary Material).

### 2.2. Procedures

The dataset was collected prospectively and included variables such as sex, age, body mass index (BMI) obtained as weight (kg) divided by height (m^2^), tumor–node–metastasis (TNM) stage according to the American Joint Committee on Cancer (AJCC), and biochemical findings such as serum albumin levels and prealbumin levels. Malnutrition was assessed using the Global Leadership Initiative on Malnutrition [GLIM] criteria [2]. To evaluate malnutrition according to these criteria, patients were required to meet at least one of the phenotypic criteria (low BMI, uncontrolled body weight loss, or low muscle mass) and one of the etiological criteria (poor food intake or assimilation, and inflammatory status or disease burden). In this study, patients with colorectal cancer were considered to meet the etiological criterion for inflammation [2]. Involuntary weight loss was defined as a loss exceeding 5% within the last 6 months or more than 10% beyond 6 months. Low BMI was defined as less than 18.5 kg/m^2^ for individuals under 70 years old or less than 20 kg/m^2^ for those over 70. Decreased muscle mass was identified by an L3-SMI of less than 40.8 cm^2^/m^2^ in men and less than 34.9 cm^2^/m^2^ in women in accordance with the GLIM-CT criteria [11]. Additionally, poor muscle mass was classified utilizing the GLIM-BIA criteria based on the European Working Group on Sarcopenia in Older People 2 (EWGSOP2) standards (<7.0 kg/m^2^ for male and <5.5 kg/m^2^ for female) [5].

### 2.3. Anthropometric and BIA-Based Skeletal Muscle Mass

Body height [cm] and waist perimeter [cm] were determined with a measuring tape [Omrom, Los Angeles, CA, USA]. Body weight was evaluated with the patients unclothed, utilizing a digital scale [Omrom, Los Angeles, CA, USA]. Using both parameters, body mass index [BMI] was calculated [body weight [kg] divided by height^2^ [m^2^]]. The EFG BIA 101 [Akern, Pisa, Italy] was used to investigate body composition. Skeletal muscle mass [SMM] was determined by bioimpedance with a precision of 5 g, using the following equation for its calculation: [0.756 Height^2^/Resistance] + [0.110 × Body mass] + [0.107 × Reactance] – 5463 [12]. SMM divided by height^2^ [m^2^] generated the skeletal muscle index [SMI-BIA]. The BIA technique was performed on the patients after 10 h of fasting, without alcohol or smoking and without prior physical exercise, between 8 and 9 in the morning. The patients did not receive any intravenous fluid infusion or enteral nutrition by tube.

### 2.4. CT-Based Skeletal Muscle Mass Measurement

The recorded variables at the L3 levels [CT, General Electric Revolution, Cincinnati, OH, USA] included the following: skeletal muscle mass area [SMA] in cm^2^, skeletal muscle area index [L3-SMI] in cm^2^/m^2^, and the average Hounsfield Unit [HU] value for each segmented tissue. Skeletal muscle volume (L) was estimated using the regression equation from Shen et al. (0.166 * L3 muscle area [cm^2^] + 2.142) [13]. Subsequently, skeletal muscle mass based on CT (SMMCT, kg) was determined by multiplying the volume of skeletal muscle by 1.06 kg/L, the standard density of skeletal muscle tissue. Furthermore, SMMCT was divided by height squared to derive the skeletal muscle index (SMI-CT, kg/m^2^). The CT scans focusing on the third lumbar vertebra [L3] were analyzed using FocusedON-BC software version 1.0 [ARTIS Development, Las Palmas Gran Canaria, España]. This program has an interface and a semiautomatic labeling device that permits user adjustments to the body mass segmentation. To measure skeletal muscle, cross-sectional CT images at Lumbar 3 were evaluated with this software. The muscles evaluated included the psoas, erector spinae, quadratus lumborum, transversus abdominis, external obliques, internal obliques, and finally rectus abdominis. Adipose tissue was categorized into subcutaneous, visceral, and intramuscular. All areas were measured in cm^2^. Tissue quality was determined by its mean Hounsfield Unit [HU] value, using the following normal cut-off points: −29 to 150 HU for muscle mass. Finally, skeletal muscle gauge [SMG] was determined by multiplying the L3-SMI value and the HU value, as suggested by Weinberg and colleagues [14]. For simplicity, instead of using [cm^2^ × HU/m^2^] as the SMG unit, an arbitrary unit [AU] was utilized.

### 2.5. Biochemical Parameters

At the time of performing the baseline nutritional evaluation with BIA, a venous blood extraction was performed, with the objective of determining the following variables: albumin [3.5–4.4 g/dL] and prealbumin [18–27 mg/dL] [Hitachi, ATM, Manheim, Germany].

### 2.6. Statistical Analysis

Normal parameters were presented as mean (standard deviation) (SD) and evaluated using independent *t*-tests for those following a normal distribution, as determined by the Kolmogorov–Smirnov test. Non-parametric parameters were examined using the Mann–Whitney U-test. Categorical parameters were expressed as frequencies and evaluated with the chi-squared test. Pearson’s correlation test was employed to evaluate the relationship between SMI-CT and SMI-BIA. Paired Student’s *t*-test was performed to assess differences between SMI values obtained via BIA and CT. Independent *t*-tests were also conducted to identify significant differences in SMI bias as measured by BIA and CT across various groups, including sex, age, BMI categories, and the presence or absence of GLIM-defined malnutrition. Cohen’s Kappa test was used as statistical test to measure the inter-rater agreement for categorical items [GLIMCT vs. GLIMBIA] and low muscle mass [CT vs. BIA criteria] [<0.20: poor agreement, 0.21–0.40: medium agreement, 0.41–0.60: moderate agreement, 0.61–0.80: good agreement, and 0.81–1.00: almost perfect agreement]. It was used to evaluate the consistency or agreement between two Bland–Altman analyses, which were used to evaluate the agreement between the two methods, with 95% limits of agreement [LOAs] calculated. Normally, acceptable agreement was indicated as a 95% limit of agreement (LOA) for bias within ±10%. The area under the receiver operating characteristic curve (AUC) was used to assess BIA’s effectiveness in detecting low muscle mass, with the optimal cut-off value determined by a better Youden index (sensitivity + specificity − 1). Data analysis was performed with SPSS software (version 23.0, Chicago, IL, USA), with statistical significance at *p*-value < 0.05.

## 3. Results

### 3.1. Patient Characteristics and Malnutrition according to GLIM Criteria

For the 137 patients with colorectal cancer [101 with colon cancer and 36 with rectal cancer], their epidemiological and anthropometric characteristics are presented in Table 1. The TNM stage distribution was as follows: Stage I with 94 patients [68.6%], Stage II with 11 patients [8.0%], and Stage III with 34 patients [24.4%]. The median age was 69.8 ± 9.5 years, with patient ages ranging from 55 to 89 years. The cohort included 88 male patients [64.2%] and 49 female patients [35.8%]. Male patients exhibited significantly higher values of SMI-L3, SMI-CT, SMI-BIA, and SMG compared to female patients, with no significant differences detected in the other variables analyzed (Table 1). The diagnosis of malnutrition according to the GLIM criteria was dependent on the method used to assess muscle mass. While more than one-third of the patients were categorized as malnourished based on the GLIM-CT data using L3-SMI [36.5%] [defined as less than 40.8 cm^2^/m^2^ in males and less than 34.9 cm^2^/m^2^ in females], a smaller proportion of patients were identified as malnourished according to GLIM-BIA using SMI-BIA [19.0%] [defined as <7.0 kg/m^2^ for men and <5.5 kg/m^2^ for women] [*p* = 0.02]. Additionally, the ability to detect patients with poor muscle mass was higher with CT [38.0%] compared to BIA [13.1%] [*p* = 0.01].

Patients diagnosed with malnutrition using the GLIM criteria, with SMI-BIA as the phenotypic criterion, exhibited significantly lower levels of albumin, prealbumin, SMI-L3, SMI-CT, SMI-BIA, and SMG compared to well-nourished patients. Similarly, significant differences in these variables were observed when comparing malnourished patients diagnosed according to the GLIM criteria using the SMI-CT phenotypic criterion with well-nourished patients (Table 2). The concordance between the two methods in identifying malnutrition [GLIM-BIA vs. GLIM-CT] was low [K = 0.300, *p* < 0.001].

### 3.2. Concordance Analysis between BIA-Defined and CT-Defined Low Muscle Mass

Using CT evaluation, low muscle mass (low SMI-L3) was diagnosed in 52 patients (38.0%), whereas the BIA identified only 18 patients (13.1%) with low muscle mass (low SMI-BIA). Among those diagnosed with low muscle mass by CT, just 29.4% (15 out of 52) were accurately identified by BIA as having low muscle mass. Conversely, 83.3% [15 out of 18] of patients identified by BIA as not having low muscle mass were rightly categorized as such when compared to the CT findings. Patients with low muscle mass as determined by SMI-BIA exhibited significantly lower levels of albumin, prealbumin, SMI-L3, SMI-CT, SMI-BIA, and SMG compared to those with normal SMI-BIA. Additionally, significant differences in SMI-L3, SMI-CT, SMI-BIA, and SMG were observed when comparing patients with low SMI-CT to those with normal SMI-CT (Table 3). The agreement between the two techniques in detecting low muscle mass [low SMI-BIA vs. low SMI-CT] was poor [K = 0.293, *p* < 0.001], with a sensitivity of 29.1%, specificity of 96.3%, accuracy of 70.0%, positive predictive data of 83.3%, and negative predictive data of 68.9%.

### 3.3. Relationship and Concordance between SMI Measurements Obtained by BIA and CT

**Figure 1:**
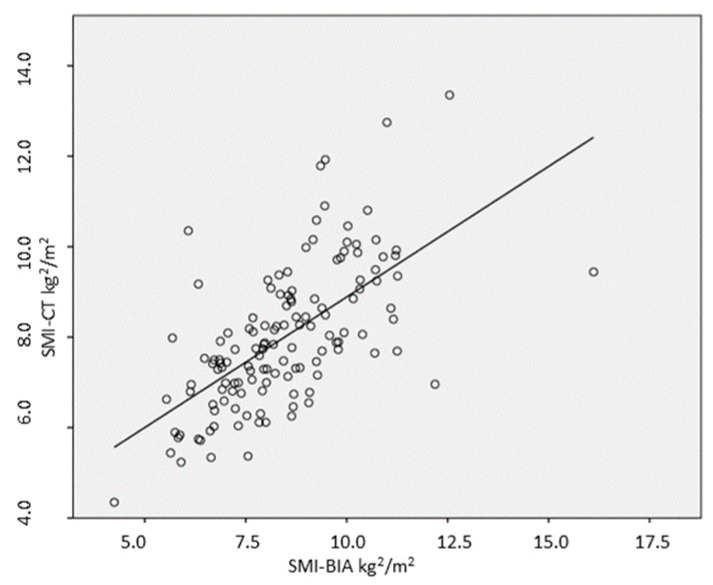
Pearson correlation between SMI values as measured by BIA and CT.

**Figure 2:**
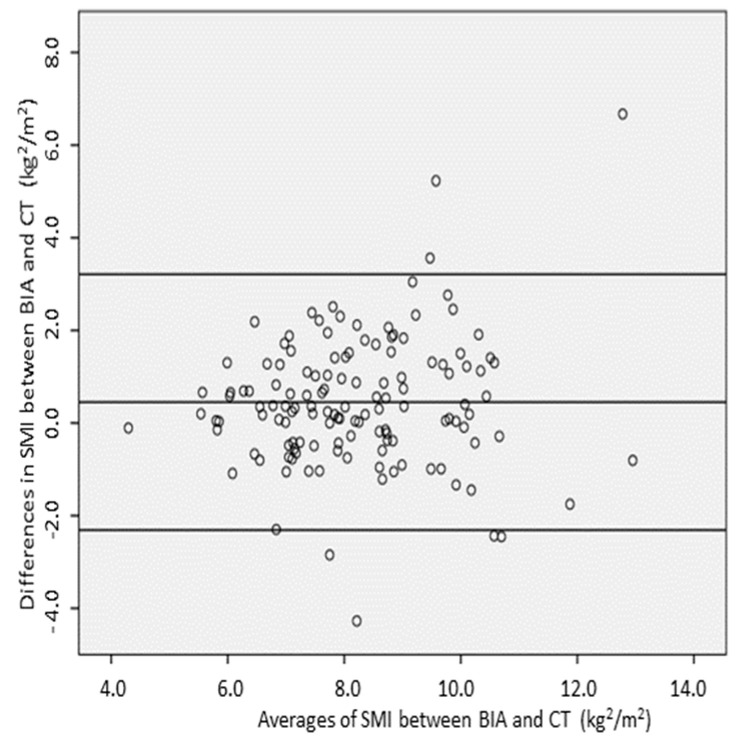
Bland–Altman plot illustrating the total differences in kilograms in the investigation of the agreement between measurements obtained by CT and BIA.

The SMI showed a positive correlation with SMI-CT in all patients (r = 0.63, *p* < 0.001, Figure 1). However, the SMI values obtained by BIA were significantly higher than those determined by CT (8.5 ± 1.7 kg/m^2^ versus 7.9 ± 1.5 kg/m^2^; *p* < 0.001). The Bland–Altman analysis revealed a substantial mean bias of 0.45 ± 1.41 kg/m^2^ (95% CI 0.21–0.70; *p* < 0.001) between SMI-BIA and SMI-CT, showing that BIA overestimated SMI by an average of 0.45 kg/m^2^ compared to CT (Figure 2). The Bland–Altman analysis further demonstrated a percentage mean bias of 12.6%, with 95% limits of agreement, and the values of bias ranged from 10.8% to 14.6%, which exceeded the clinically acceptable threshold of 10%. This suggests that the bias level is too high for reliable clinical use, indicating that the two measurement methods are not directly interchangeable. Although the differences between SMI-BIA and SMI-CT fell within the clinically acceptable range for 61 patients (45.9%), BIA overestimated SMI compared to CT in 72 patients (54.1%) and did not underestimate SMI in any patients.

Table 4 presents a comparative analysis of SMI differences across various subgroups, including sex, age, BMI, low muscle mass, and GLIM-defined malnutrition. The analysis revealed significant differences in the mean bias between the SMI measurements obtained by CT and BIA, particularly when comparing patients with and without malnutrition, as well as those with and without low muscle mass. Specifically, there was a significant difference in the mean bias of SMI determined by BIA [0.58 ± 1.2 kg/m^2^ vs. 0.39 ± 1.5 kg/m^2^; *p* = 0.03] and by CT [0.59 ± 1.4 kg/m^2^ vs. −0.06 ± 1.3 kg/m^2^; *p* = 0.01] between malnourished and well-nourished patients according to the GLIM-CT criteria, indicating that BIA significantly overestimated SMI in patients with GLIM-defined malnutrition (Table 5).

### 3.4. SMI-BIA for Detecting Low Muscle Mass

**Figure 3:**
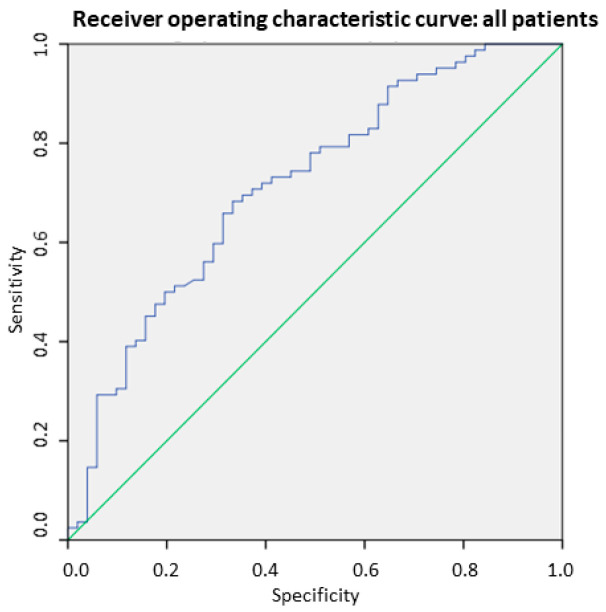

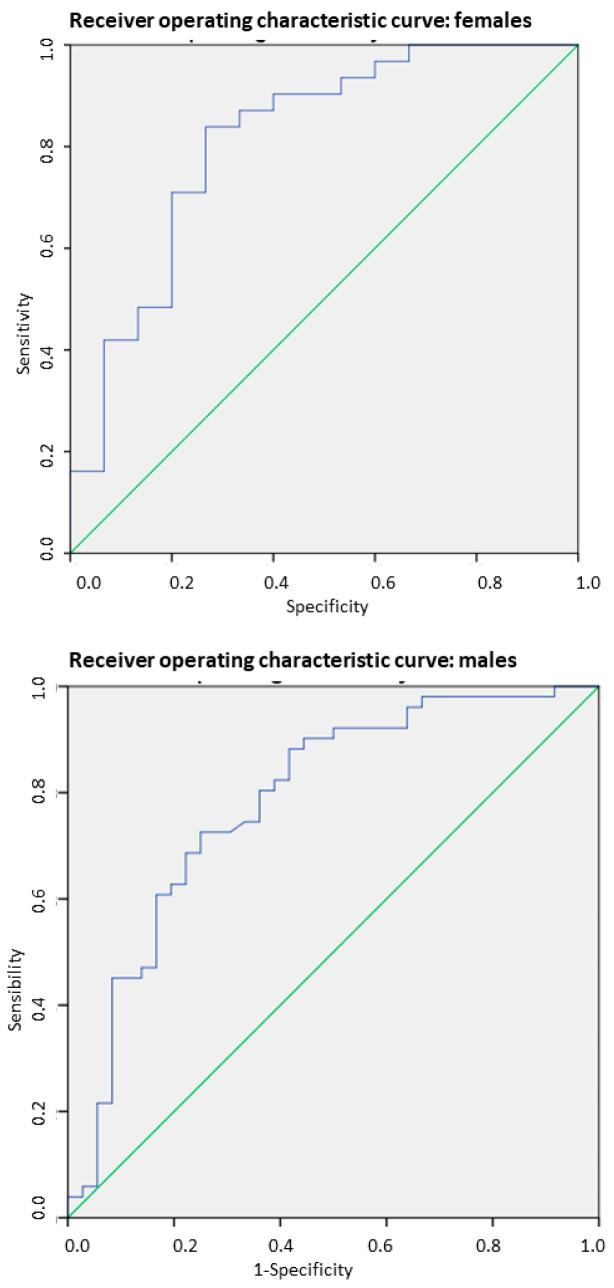
Receiver operating characteristic (ROC) curve analysis was conducted to investigate the effectiveness of BIA-derived SMI in detecting CT-defined low muscle mass across the entire group, as well as separately for males and females.

As illustrated in Figure 1, SMI-BIA also showed a good correlation with L3-SMI [r = 0.63; *p* = 0.001], with this correlation observed in both males [r = 0.54; *p* = 0.001] and females [r = 0.57; *p* = 0.001]. However, SMI-BIA values were significantly lower in patients with CT-defined poor muscle mass [7.5 ± 1.4 kg/m^2^ vs. 9.0 ± 1.2 kg/m^2^; *p* < 0.001], with similar differences noted in both male [8.5 ± 1.6 kg/m^2^ vs. 9.2 ± 1.67 kg/m^2^; *p* = 0.001] and female patients [7.1 ± 1.1 kg/m^2^ vs. 7.3 ± 1.1 kg/m^2^; *p* = 0.001]. Receiver operating characteristic (ROC) curves were constructed to evaluate the effectiveness of SMI-BIA in identifying low muscle mass, using CT as the reference standard. The area under the curve (AUC) for SMI-BIA in detecting low muscle mass was 0.714 (95% CI: 0.624–0.824), with an optimal cut-off value of 8.1 kg/m^2^, achieving a sensitivity of 68.3% and a specificity of 66.9% (Figure 3). For female patients, the AUC was 0.813 (95% CI: 0.671–0.995) with a threshold value of 6.8 kg/m^2^, resulting in a sensitivity of 83.9% and a specificity of 73.3%. For male patients, the AUC was 0.785 (95% CI: 0.683–0.881), with a threshold value of 8.9 kg/m^2^, achieving sensitivity and specificity values of 72.5% and 75.0%, respectively.

## 4. Discussion

Having a standardized tool for diagnosing malnutrition is crucial, particularly in patients with abdominal cancer, who are at a heightened risk for disease-related malnutrition [DRM], which can worsen their condition perioperatively and increase the likelihood of complications. The Global Leadership Initiative for Malnutrition (GLIM) criteria encompass the evaluation of muscle mass, a factor not typically included in other malnutrition diagnostic tools [2]. The GLIM criteria propose several techniques for assessing muscle mass, including dual-energy X-ray absorptiometry (DXA), BIA, CT, and magnetic resonance imaging (MRI). Given the local resources, we opted for routine CT, which is consistently available for gastrointestinal cancer patients, and BIA, a longstanding component of our nutritional assessments. Numerous studies have established significant associations between CT-defined sarcopenia and various outcomes in surgical patients [15]. BIA, a non-invasive bedside technique, facilitates straightforward body composition measurements, with the resulting muscle mass linked to clinical outcomes [16].

In our cohort of oncological patients, the malnutrition rate was significantly higher when assessed utilizing the GLIM criteria with muscle mass measured by CT [GLIM-CT] compared to BIA [GLIM-BIA]. While the malnutrition rates determined by GLIM-CT were consistent with previous studies [17], the prevalence was notably lower with BIA. Using the ESPEN guideline’s recommended cut-offs for skeletal muscle index [SMI] [2], we observed a significantly lower incidence of reduced muscle mass compared to the thresholds established for CT [11]. The ESPEN-recommended cut-offs appear stricter, depicting patients as having poorer nutritional status compared to sarcopenia defined by earlier CT cut-offs. It is plausible to theorize that BIA may overestimate skeletal muscle in colorectal cancer patients when compared to the CT reference standard, resulting in an underestimation of the prevalence of low muscle mass. This discrepancy is evident in our oncological group and clearly explains the variation in malnutrition prevalence. The bias between SMI-CT and SMI-BIA might be attributed to the predefined cut-off values and the fact that SMI-BIA is only a proxy for measuring muscle mass, which depends on electrical parameters. Additionally, considering the phase angle when defining malnutrition might be beneficial, as it has been demonstrated in other studies to be a reliable predictor of surgical complications [18].

Despite the availability of various body composition assessment techniques, a recently conducted meta-analysis and systematic review identified CT and BIA as the most frequently used methods in cancer patients [1]. BIA is advantageous due to its convenience, offering results in minutes. However, certain limitations should be acknowledged when integrating BIA into routine clinical activity. BIA is a surrogate method for estimating skeletal muscle mass, and its precision is strongly reliant on the use of a right equation [19]. Additionally, BIA results could be affected by hydration status; overhydration and edema may lead to an overestimation, whereas dehydration can lead to an underestimation of muscle mass [3]. Patients with colorectal cancer are particularly vulnerable to malnutrition and hydration fluctuations, which can alter the balance of intracellular and extracellular water. Therefore, the direct use of BIA results for body composition assessment in cancer patients remains a subject of debate. Recent comparisons of skeletal muscle mass assessed by BIA and CT in different populations have shown that the former tends to overestimate muscle mass. For example, Hassen et al. [20] found a BIA overestimation of 2.11 ± 6.11 kg/m^2^ in patients with non-small-cell lung cancer, while Kim et al. [21] reported overestimation in critically ill patients with low skeletal muscle mass. Zuo et al. [22] reported that BIA overestimated SMI by 1.18 ± 0.96 kg/m^2^ in gastric cancer patients, despite a significant association between SMI-CT and SMI-BIA. In a similar vein, BIA considerably overestimated SMI in colorectal cancer patients who were identified as malnourished based on the GLIM criteria, in comparison to those who were not classified as malnourished. Therefore, BIA tends to overestimate skeletal muscle mass relative to CT, necessitating careful interpretation of its findings in cancer patients. Recent studies, including one with 67 patients with various cancer types, have yielded results similar to our current data [23]. Additionally, Jones et al. [24] demonstrated that BIA and mid-arm muscle circumference measurements overestimated muscle mass in surgical patients with colorectal cancer.

There is an increasingly strong focus on identifying poor muscle mass in clinical practice, as it is a prevalent marker of cachexia, undernutrition, and sarcopenia, all of which are correlated with poor clinical situations [25]. Failure to accurately identify patients with these conditions can delay appropriate interventions, potentially worsening their clinical prognosis. Sarcopenia as defined by BIA has been found to be significantly related with adverse clinical events in cancer patients, including decreased total survival rate and increased surgical complications [26]. Therefore, precise detection of poor muscle mass in cancer patients is essential due to its substantial impact on prognosis. The European Working Group on Sarcopenia in Older People 2 [EWGSOP2] and the GLIM Body Composition Working Group have endorsed BIA as a suitable tool for evaluating muscle mass [2,11]. Nevertheless, as previously mentioned, BIA may significantly underestimate the prevalence of poor muscle mass when contrasted with CT scans. For instance, Kikuchi et al. [27] found that, in patients with liver disease, the occurrence of reduced muscle mass was around 20% with BIA and 48% with CT based on their respective cut-offs. In our present study, when applying the BIA-measured SMI thresholds for poor muscle mass as indicated by EWGSOP2, the occurrence of poor muscle mass was merely 13.1%, a percentage that was markedly lower than the 38.0% identified by CT. The concordance between the two techniques for evaluating reduced muscle mass was minimal. In this study, the BIA-measured SMI thresholds were set at 8.9 kg/m^2^ for men and 6.8 kg/m^2^ for women. When muscle mass as determined by BIA falls below these values, it should arouse doubt of poor muscle mass, thus assisting in accurately classifying patients in clinical settings where CT scans are not accessible.

Specific studies in different pathologies are required to determine SMI-BIA cut-off points that do not underestimate sarcopenia and malnutrition when using general cut-off points [2]. A significant gap exists in standardized threshold data for detecting low muscle mass. The GLIM Body Composition Working Group highlights the importance of establishing cut-offs that account for race, gender, age, and disease-specific status [2]. According to the EWGSOP2 guidelines, BIA-measured SMI cut-offs for diagnosing sarcopenia are <7.0 kg/m^2^ for men and <5.7 kg/m^2^ for women [11]. However, standardized cut-off values for assessing poor muscle mass via CT are still not well established. Martin et al. have proposed widely accepted CT thresholds for low muscle mass at the Lumbar 3 vertebra across various cancers: <43 cm^2^/m^2^ for men with a BMI < 25 kg/m^2^, <53 cm^2^/m^2^ for men with a BMI ≥ 25 kg/m^2^, and <41 cm^2^/m^2^ for women, regardless of BMI [28,29,30,31]. In our study, we utilized the L3 threshold values of ≤40.8 cm^2^/m^2^ for men and ≤34.9 cm^2^/m^2^ for women, which were derived from a cohort of healthy Caucasian individuals [11]. While these values are common in other studies, they may not be applicable to other ethnicities or different cancers. Moreover, additional investigation is essential to establish suitable cut-off values for identifying low muscle mass in cancer patients, keeping factors such as age, sex, and race in mind across various measurement techniques. At present, knowledge on the nutritional evaluation of patients with colorectal cancer is an area of great interest due to the identification of new biological markers [32] and anthropometric parameters [33], as well as the emergence of technology based on artificial intelligence to evaluate the images obtained using classic techniques such as abdominal CT [34,35]. It is also necessary to take into account that colorectal surgery has complications in many cases that are related to the nutritional status of patients [36], suture dehiscence, fistulas, infections, and hemorrhages. Therefore, nutritional evaluation of these patients with artificial intelligence techniques applied to CT images obtained during clinical follow-up is an area of interest to develop personalized medication for these patients.

Our study has several limitations. Firstly, we did not assess physical performance, such as timed up-and-go test or handgrip strength. The absence of these indicators may limit the study’s ability to comprehensively evaluate the nutritional and health status of the patients and may overlook the potential importance of muscle function in the diagnosis of malnutrition. Moreover, previous investigations have demonstrated that the diagnostic method employed can impact the proportion of patients identified with low muscle strength [32]. We did not assess the presence of edema in our patients, which may have influenced the precision of BIA determinations for skeletal muscle mass. In addition, our study may have different biases in relation to the method of patient selection (selection biases). Our study may also have problems related to the lack of matching, as well as the presence of uncollected variables that could potentially modify the size of the effects described. One of these potential factors is hydration status; as BIA results could be affected by hydration status, overhydration and edema could lead to an overestimation, whereas dehydration could result in an underestimation of muscle mass. Moreover, this study was conducted at a single medical center, which may generate sample selection bias and decrease the generalizability of the study findings. Finally, it is important to note that only the Akern device was utilized for BIA assessment, and different devices may produce varying measurements of skeletal muscle mass. The strengths of our design include a homogeneous population of patients with colorectal cancer and prospectively collected data using BIA for all patients. New parameters, such as skeletal muscle index [SMI] and radiodensity [HU], are well-evaluated prognostic factors in cancer patients. The impact of an integrated determination, called the skeletal muscle gauge [SMG], has been evaluated in only one previous study [37] in patients with colorectal cancer. Our current study confirms the strong association of this surrogate parameter with SMI and malnutrition classification in these patients.

## 5. Conclusions

In conclusion, despite the strong correlation, skeletal muscle mass measurements obtained from BIA and CT are not interchangeable. BIA produces an overestimation of skeletal muscle mass in colorectal cancer patients when compared to CT. Using the BIA-measured SMI thresholds recommended by the European Working Group on Sarcopenia in Older People 2 (EWGSOP2) for detecting poor muscle mass raises concerns that BIA may underestimate the prevalence of poor muscle mass and malnutrition in these patients. Therefore, further research is required to generate appropriate threshold values for identifying poor muscle mass in oncological patients. The current evidence indicates that CT offers greater precision in assessing muscle mass in patients with colorectal tumor [38]. Variations in equipment, operational methods, and calculation formulas can result in discrepancies in outcomes. Future research should examine how standardization across different devices and operational protocols can impact measurement consistency, allowing for more precise comparisons between various techniques.

## Figures and Tables

**Table 1:** Patient clinical and biochemical characteristics.

Parameters	All	Male	Female	*p*-Value
Age, years	69.8 ± 9.5	69.3 ± 9.4	70.6 ± 9.8	0.36
Gender (female/male)	49/88	88	49	0.03
Albumin, g/L	4.3 ± 0.4	4.4 ± 0.3	4.3 ± 0.5	0.41
Prealbumin, mg/dL	25.2 ± 7.5	26.7 ± 7.5	23.3 ± 7.1	0.38
L3-SMI, cm^2^/m^2^	40.3 ± 8.9	43.7 ± 8.4	34.6 ± 7.5	0.02
SMI-BIA, kg^2^/m^2^	8.5 ± 1.7	9.1 ± 1.7	7.3 ± 1.1	0.03
SMI-CT, kg^2^/m^2^	7.9 ± 1.5	8.4 ± 1.5	7.1 ± 1.1	0.02
SMG, AU	1511.5 ± 588.5	1689.2 ± 597.5	1189.6 ± 425.5	0.01
GLIM-BIA malnourished, %	19.0%	18.2%	20.8%	0.56
GLIM-CT malnourished, %	36.5% *	36.4%	38.3%	0.49
CT-defined low muscle mass, %	38.0%	40.9	32.7	0.23
BIA-defined low muscle mass, %	13.1% **	16.1%	8.7%	0.15

[*] *p* = 0.02 for diagnosis of malnourished based on GLIM-BIA vs. GLIM-CT. [**] *p* = 0.01 for CT-defined poor muscle mass vs. BIA-defined poor muscle mass.

**Table 2:** Patient clinical and biochemical parameters for malnutrition diagnosis based on GLIM-BIA and GLIM-CT.

Parameters	GLIM-BIA Well-Nourished	GLIM-BIA Malnutrition	*p*-Value	GLIM-CT Well-Nourished	GLIM-CT Malnutrition	*p*-Value
Age, years	68.6 ± 9.4	74.6 ± 8.9	0.01	68.4 ± 4.2	70.2 ± 9.7	0.20
Gender (female/male)	38/72	11/16	0.43	34/53	15/35	0.38
Albumin, g/L	4.4 ± 0.3	3.9 ± 0.2	0.01	4.3 ± 0.3	4.1 ± 0.4	0.04
Prealbumin, mg/dL	26.4 ± 3.1	20.0 ± 3.0	0.02	26.3 ± 2.9	24.6 ± 2.6	0.04
L3-SMI, cm^2^/m^2^	41.9 ± 8.6	33.6 ± 7.6	0.01	43.7 ± 8.4	34.6 ± 7.5	0.02
SMI-BIA, kg^2^/m^2^	8.9 ± 1.5	6.7 ± 1.1	0.02	9.1 ± 1.6	7.5 ± 1.4	0.02
SMI-CT, kg^2^/m^2^	8.2 ± 1.4	6.6 ± 1.1	0.02	8.8 ± 1.5	6.9 ± 1.1	0.01
SMG, AU	1596.3 ± 581.5	1158.9 ± 484.5	0.01	1700.8 ± 517.5	1244.9 ± 424.5	0.01
CT-defined poor muscle mass (%)	29.1%	76.9%	0.001	0%	86.7%	0.001
BIA-defined poor muscle mass (%)	0%	69.2%	0.001	4.1%	25.9%	0.001

**Table 3:** Patient clinical and biochemical characteristics based on low muscle mass as determined by CT and BIA.

Parameters	Low SMI-CT	Normal SMI-CT	*p*-Value	Low SMI-BIA	Normal SMI-BIA	*p*-Value
Age	69.4 ± 9.4	70.2 ± 9.3	0.48	76.3 ± 6.5	68.7 ± 5.8	0.26
Gender (female/male)	16/36	33/52	0.29	7/15	42/73	0.03
Albumin, g/L	4.2 ± 0.4	4.3 ± 0.4	0.48	3.9 ± 0.4	4.4 ± 0.4	0.02
Prealbumin, mg/dL	24.7 ± 6.6	26.1 ± 6.9	0.37	20.3 ± 7.4	26.1 ± 7.2	0.01
L3-SMI, cm^2^/m^2^	34.5 ± 6.4	43.7 ± 5.5	0.02	33.9 ± 7.8	41.4 ± 8.1	0.01
SMI-BIA, kg^2^/m^2^	7.5 ± 1.4	9.0 ± 1.2	0.03	6.5 ± 0.9	8.7 ± 1.0	0.02
SMI-CT, kg^2^/m^2^	6.9 ± 1.1	8.6 ± 1.5	0.01	6.8 ± 1.3	8.2 ± 1.1	0.02
SMG, AU	1689.8 ± 599.5	1171.8 ± 383.5	0.01	1579.1 ± 424.1	1203.1 ± 510.8	0.01
GLIM-BIA malnourished (%)	38.5%	7.1%	0.02	100%	7.0%	0.001
GLIM-CT malnourished (%)	100%	9.6%	0.001	25.9%	74.1%	0.02

**Table 4:** Analysis of mean values and Pearson correlation for SMI as determined by CT and BIA.

Group	*n*	SMIBIA (kg/m^2^)	SMICT (kg/m^2^)	*p*-Value	r	*p*-Value
All	137	7.9 ± 1.5	8.4 ± 1.7	0.001	0.63	0.001
Female	49	7.3 ± 1.1	7.1 ± 1.1	0.001	0.57	0.001
Male	88	9.0 ± 1.7	8.5 ± 1.6	0.001	0.54	0.001
<65 y	41	9.4 ± 1.6	8.5 ± 1.8	0.001	0.64	0.001
>65 y	96	8.1 ± 1.6	7.6 ± 1.3	0.001	0.61	0.001
BMI < 25	34	7.2 ± 1.5	6.9 ± 1.0	0.001	0.57	0.001
BMI > 25	103	9.0 ± 1.6	8.3 ± 1.5	0.001	0.59	0.001
GLIM-CT well-nourished	84	9.0 ± 1.7	8.6 ± 1.5	0.001	0.53	0.001
GLIM-CT malnourished	53	7.5 ± 1.4	6.9 ± 1.1	0.001	0.59	0.001
GLIM-BIA well-nourished	106	8.9 ± 1.6	8.3 ± 1.5	0.001	0.57	0.001
GLIM-BIA malnourished	31	6.9 ± 1.3	6.8 ± 1.5	0.23	0.51	0.009
CT-defined normal muscle mass (%)	82	8.9 ± 1.7	8.8 ± 1.5	0.39	0.58	0.001
CT-defined low muscle mass (%)	55	7.7 ± 1.5	6.7 ± 0.9	0.001	0.60	0.001
BIA-defined normal muscle mass (%)	115	8.7 ± 1.6	8.2 ± 1.5	0.001	0.61	0.001
BIA-defined low muscle mass (%)	22	6.8 ± 1.3	6.4 ± 0.9	0.21	0.45	0.049

In all subgroups, SMI-BIA shows a positive correlation with SMI-CT, and SMI-BIA was found to be significantly higher than SMI-CT, except in patients with poor muscle mass as determined by BIA and with normal muscle mass as determined by CT.

**Table 5:** Bland–Altman assessment of SMI measurements obtained by CT and BIA.

Group	*n*	SMIBIA-CT (kg/m^2^)	*p* *	95% LOA
All	137	0.45 ± 1.4	-	0.21–0.65
Female	49	0.23 ± 1.0	-	−0.06–0.52
Male	88	0.60 ± 1.6	0.19	0.23–0.90
<65 y	41	0.85 ± 1.4	-	0.39–1.30
>65 y	96	0.27 ± 1.4	0.12	0.008–0.55
BMI < 25	34	0.24 ± 1.3	-	0.68–0.20
BMI > 25	103	0.69 ± 1.4	0.15	0.41–0.97
GLIM-CT well-nourished	84	0.39 ± 1.1	-	0.06–0.72
GLIM-CT malnourished	53	0.58 ± 0.9	0.01	0.24–0.92
GLIM-BIA well-nourished	106	−0.06 ± 1.3	-	−0.56–0.44
GLIM-BIA malnourished	31	0.59 ± 1.4	0.03	0.31–0.87
CT-defined normal muscle mass (%)	82	0.13 ± 1.4	-	−0.17–0.44
CT-defined low muscle mass (%)	55	0.96 ± 1.3	0.001	0.61–1.31
BIA-defined normal muscle mass (%)	115	0.58 ± 1.4	-	0.33–0.84
BIA-defined low muscle mass (%)	22	−0.39 ± 1.3	0.006	−1.02–0.24

*p* *: differences in bias of SMI as determined by CT and BIA.

## Data Availability

The data presented in this study are available on request from the corresponding author. Data are not publicly due to privacy or ethical restrictions.

## Supplementary Materials

The following supporting information can be downloaded at: https://www.mdpi.com/article/10.3390/nu16173035/s1, File S1: Strobe statement.
